# Supplementary material for: Diversity and evolution of a phase-variable multi-locus antigen in Neisseria gonorrhoeae
Source: bioRxiv. 2026 Feb 5:2026.02.02.703239. Preprint. [Version 2] doi: 10.64898/2026.02.02.703239 (PMC12889595; doi:10.64898/2026.02.02.703239)
Supplement: Supplement 5 [file NIHPP2026.02.02.703239v2-supplement-5.pdf]

bioRxiv preprint doi: https://doi.org/10.64898/2026.02.02.703239; this version posted February 5, 2026. The copyright holder for this preprint (which was not certified by peer review) is the author/funder, who has granted bioRxiv a license to display the preprint in perpetuity. It is made available under aCC-BY 4.0 International license.

Supplementary figures

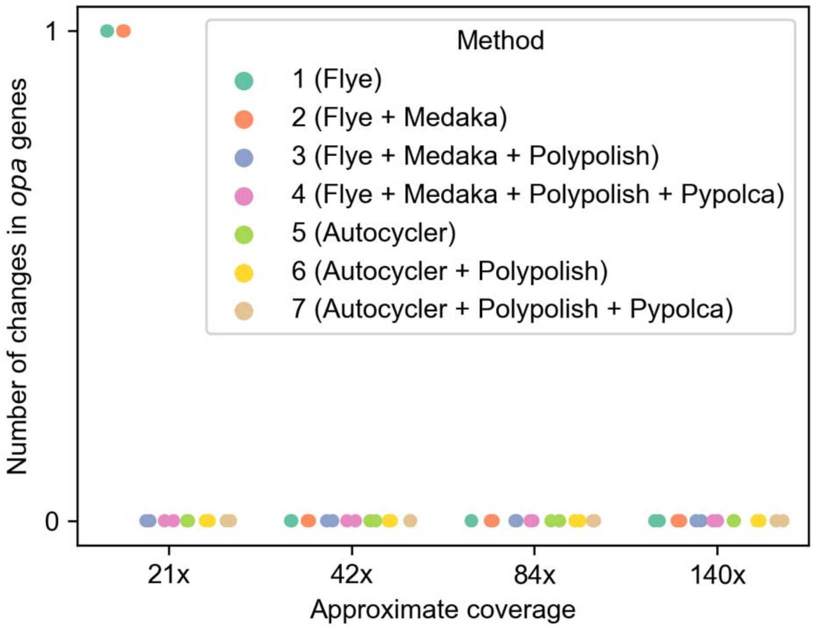

Figure S 1: Comparison of *opa* sequences across long-read assembly and polishing methods. The number of changes in *opa* genes at 4 read coverage levels using 7 different assembly and polishing procedures. There are two points of the same color in each coverage level, indicating two different isolates. The changes at 21x for methods 1 and 2 include one SNP in one isolate’s genome and one single base insertion in the other isolate’s genome.

bioRxiv preprint doi: https://doi.org/10.64898/2026.02.02.703239; this version posted February 5, 2026. The copyright holder for this preprint (which was not certified by peer review) is the author/funder, who has granted bioRxiv a license to display the preprint in perpetuity. It is made available under aCC-BY 4.0 International license.

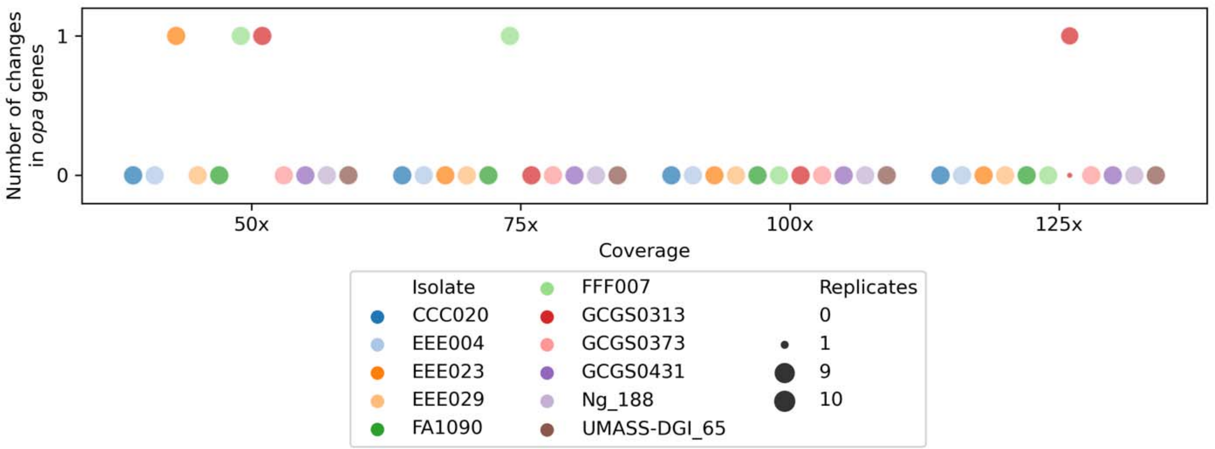

Figure S 2: More extensive comparison of *opa* sequences in Autocycler assemblies across 11 diverse isolates at 4 read coverage levels. The number of changes in *opa* genes at 4 read coverage levels using Autocycler. For each isolate, the reads were randomly subsampled 10 times at each read coverage (replicates) and an assembly was created with the subsampled reads using Autocycler. The size of the point indicates the number of replicates. The changes at 50x coverage include one SNP in two separate genomes and one undetected *opa*, the change at 75x was multiple sequence differences in one genome, and the change at 125x coverage was one SNP in one genome.

bioRxiv preprint doi: https://doi.org/10.64898/2026.02.02.703239; this version posted February 5, 2026. The copyright holder for this preprint (which was not certified by peer review) is the author/funder, who has granted bioRxiv a license to display the preprint in perpetuity. It is made available under aCC-BY 4.0 International license.

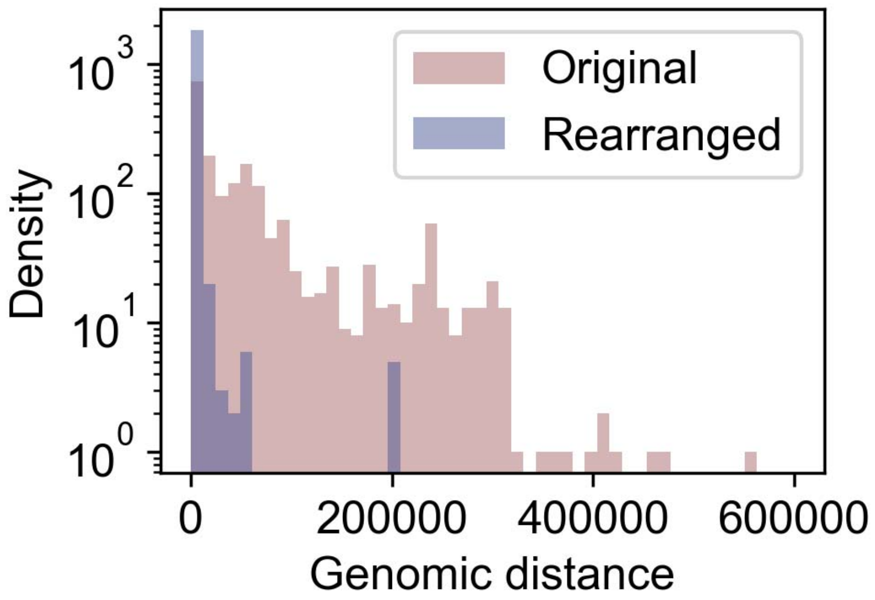

Figure S 3: **The distance between *opa* genes is higher in the original positions than after accounting for genomic rearrangements.** The distances are calculated as the distance between each *opa* in the reference genome FA1090 to the *opa* that is closest in genomic position in all other isolates that had 11 *opa*.

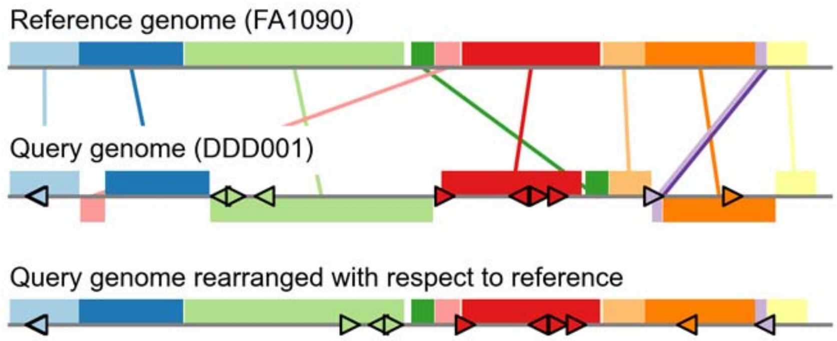

Figure S 4: Schematic of genomic rearrangement procedure (see Methods). The shaded colored regions are the locally collinear blocks (LCBs). The LCBs that appear above the gray line are on the forward strand and those that appear below the gray line are on the reverse

bioRxiv preprint doi: https://doi.org/10.64898/2026.02.02.703239; this version posted February 5, 2026. The copyright holder for this preprint (which was not certified by peer review) is the author/funder, who has granted bioRxiv a license to display the preprint in perpetuity. It is made available under aCC-BY 4.0 International license.

strand. A triangle pointing to the right indicates an *opa* gene on the forward strand and a triangle pointing to the left indicates an *opa* gene on the reverse strand.

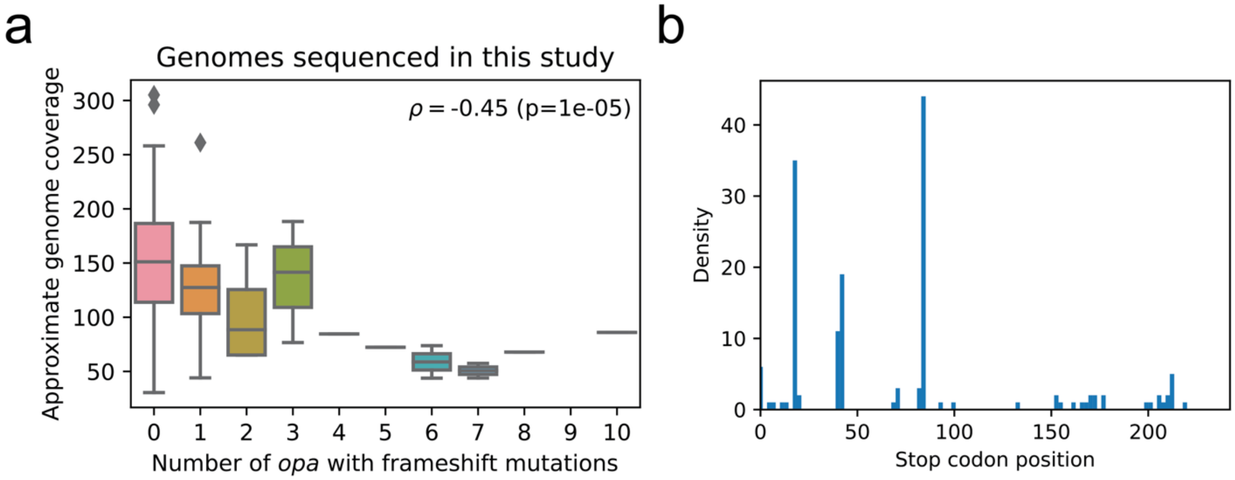

Figure S 5: A subset of *opa* genes exhibit frameshift mutations after coding repeats leading to a premature stop codon. (a) The approximate genome sequencing coverage and number of *opa* in the genome with frameshift mutations downstream of the coding repeats. (b) The locations of the stop codons in the *opa* genes with frameshift mutations downstream of the coding repeats. The maximum value of the x-axis is set at the average length of *opa* amino acid sequences.

bioRxiv preprint doi: https://doi.org/10.64898/2026.02.02.703239; this version posted February 5, 2026. The copyright holder for this preprint (which was not certified by peer review) is the author/funder, who has granted bioRxiv a license to display the preprint in perpetuity. It is made available under aCC-BY 4.0 International license.

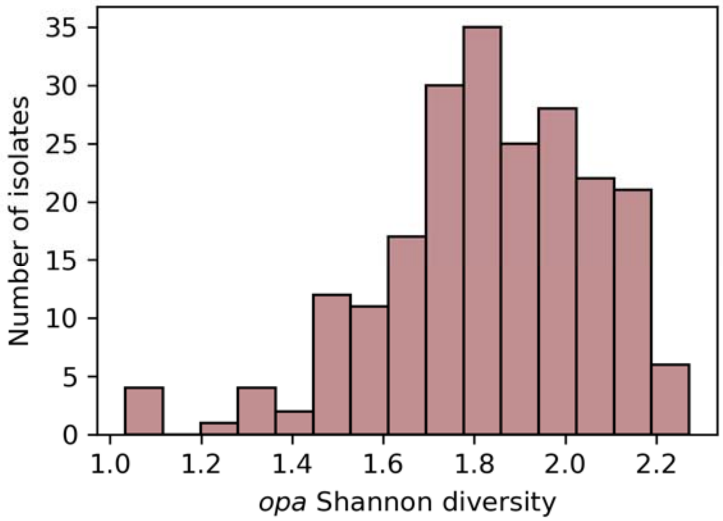

Figure S 6: Shannon diversity of *opa* types within isolates.

bioRxiv preprint doi: https://doi.org/10.64898/2026.02.02.703239; this version posted February 5, 2026. The copyright holder for this preprint (which was not certified by peer review) is the author/funder, who has granted bioRxiv a license to display the preprint in perpetuity. It is made available under aCC-BY 4.0 International license.

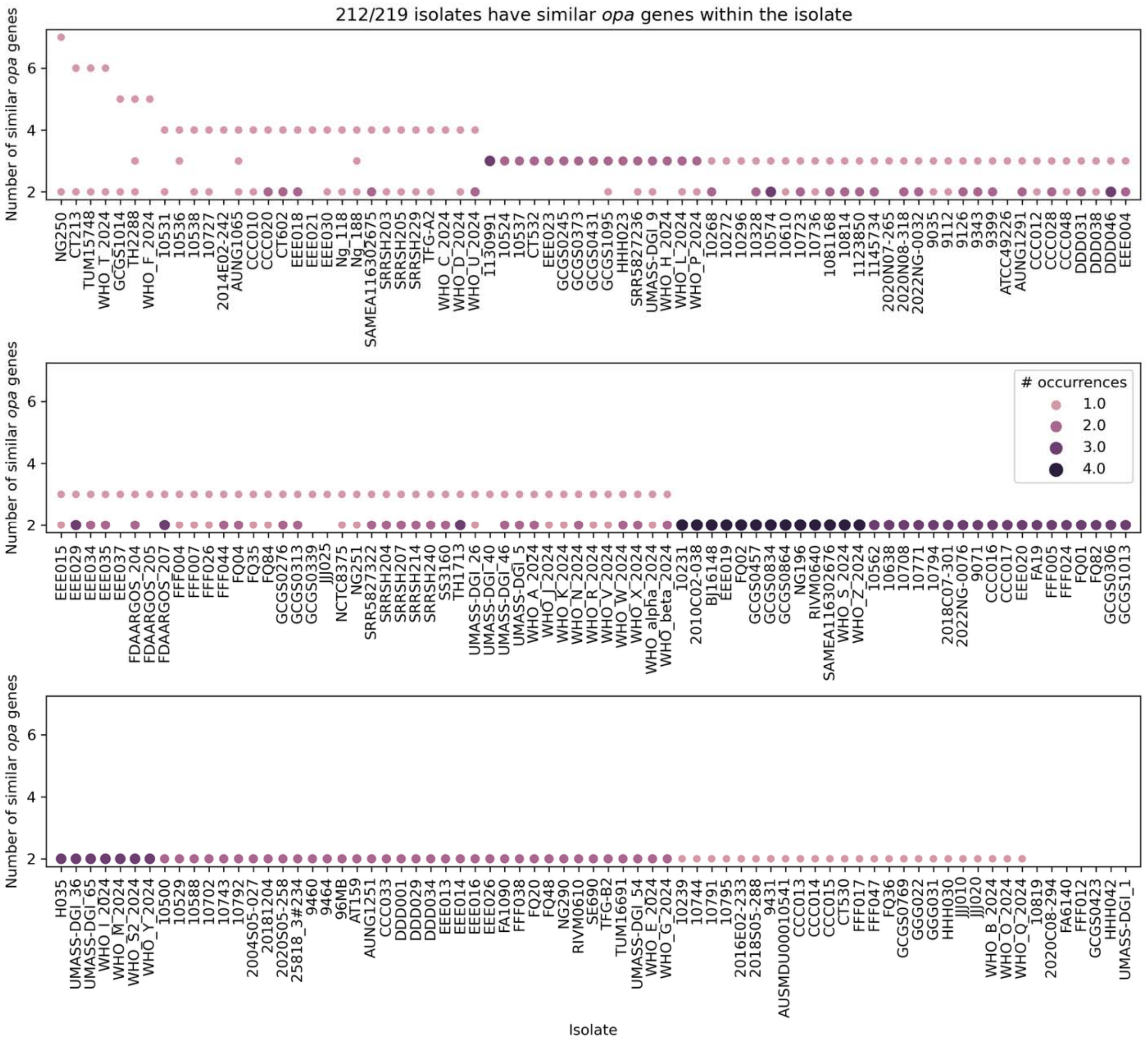

Figure S 7: **Genomes exhibit distinct patterns of similar *opas*.** All isolates with complete genomes are shown on the x-axis. The points indicate groups of *opa* genes in the same isolate with >95% amino acid sequence identity. The y-axis shows the number of similar *opa* genes in each group. The size and color of the point indicate the number of distinct groups of each size in the genome. The x-axis is sorted first by the maximum number of similar *opa* genes in any group and then by the maximum number of groups. The plot is split into three rows for readability.

bioRxiv preprint doi: https://doi.org/10.64898/2026.02.02.703239; this version posted February 5, 2026. The copyright holder for this preprint (which was not certified by peer review) is the author/funder, who has granted bioRxiv a license to display the preprint in perpetuity. It is made available under aCC-BY 4.0 International license.

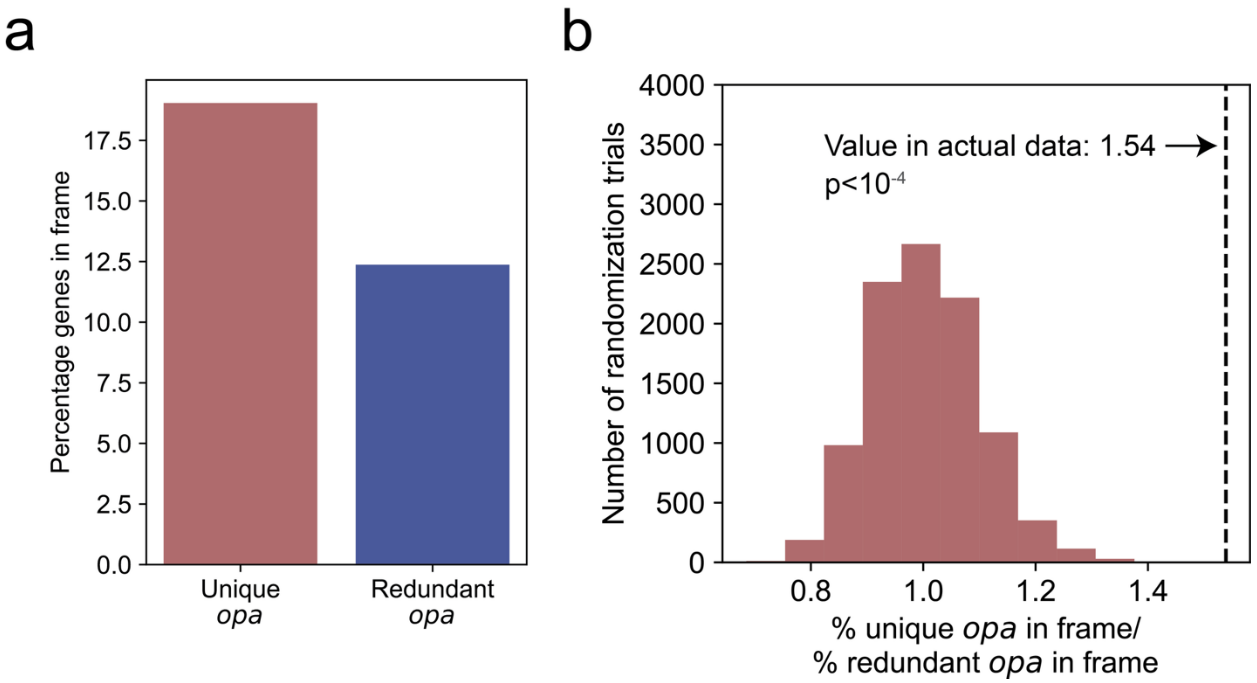

Figure S 8: The *opa* that are unique in an isolate are more likely to be in frame than the *opa* that are redundant. (a) The percentage of *opa* that are in frame for *opa* that are unique within an isolate (<95% amino acid similarity) or redundant within an isolate (≥95% amino acid similarity). (b) The ratio of the percentage of unique *opa* in frame to the percentage of redundant *opa* in frame for 10^4 randomizations of the data. The randomization procedure permuted which *opa* are labeled redundant, keeping the same total number of redundant *opa* across all isolates. Zero randomizations gave ratios as high as in the actual data (ratio of 1.54, indicated by the vertical dashed black line) yielding a p-value of less than 10^{-4}.

bioRxiv preprint doi: https://doi.org/10.64898/2026.02.02.703239; this version posted February 5, 2026. The copyright holder for this preprint (which was not certified by peer review) is the author/funder, who has granted bioRxiv a license to display the preprint in perpetuity. It is made available under aCC-BY 4.0 International license.

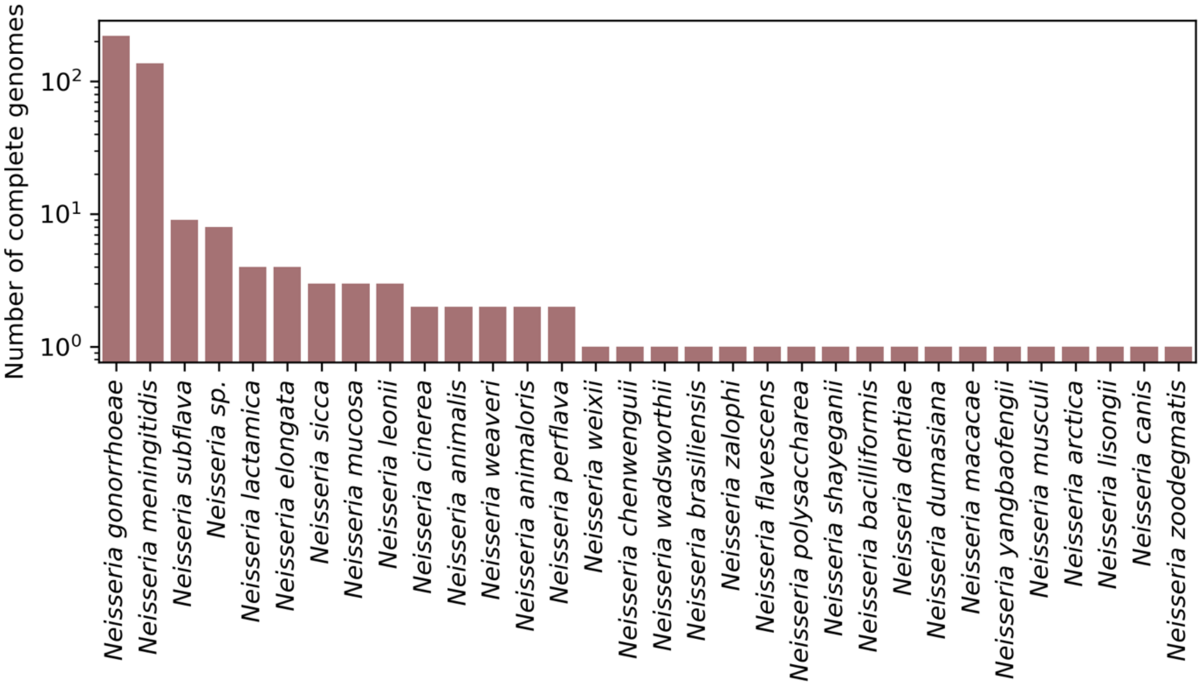

Figure S 9. The number of publicly available complete genomes by species from the *Neisseria* genus.

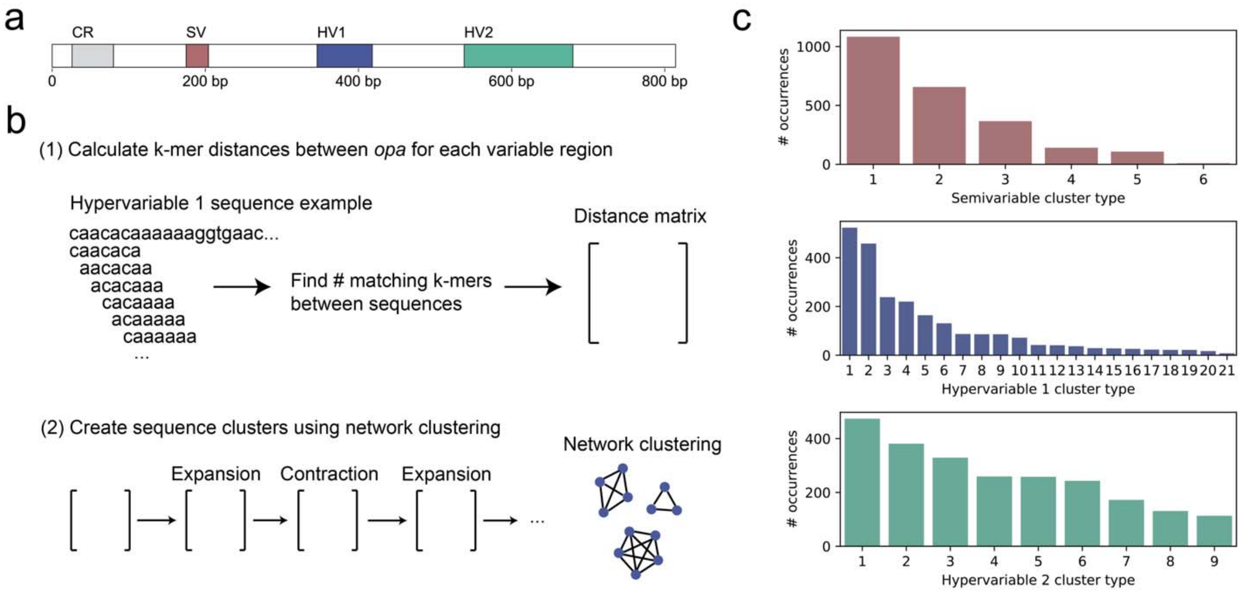

bioRxiv preprint doi: https://doi.org/10.64898/2026.02.02.703239; this version posted February 5, 2026. The copyright holder for this preprint (which was not certified by peer review) is the author/funder, who has granted bioRxiv a license to display the preprint in perpetuity. It is made available under aCC-BY 4.0 International license.

Figure S 10: Network-based clustering approach for semivariable and hypervariable *opa* sequences. (a) Schematic of the *opa* gene. The exact length and locations of the gene features varies across *opa* genes; depicted here is FA1090 *opa1*. CR: coding repeat, SV: semivariable region, HV1: hypervariable 1 region, HV2: hypervariable 2 region. (b) Summary of the approach to clustering variable region sequences. For each variable region (semivariable, hypervariable 1, and hypervariable 2), we calculated the k-mer distances between all sequences using MASH, setting k such that the probability of finding a random k-mer in each sequence is 0.01. We performed successive rounds of inflation (expansion and contraction) on the distance matrix, which amplifies high values of the matrix and suppresses low values of the matrix. We chose the lowest inflation parameter that gave a stable clustering. (c) The distribution of the cluster types for the sequences in each variable region.

bioRxiv preprint doi: https://doi.org/10.64898/2026.02.02.703239; this version posted February 5, 2026. The copyright holder for this preprint (which was not certified by peer review) is the author/funder, who has granted bioRxiv a license to display the preprint in perpetuity. It is made available under aCC-BY 4.0 International license.

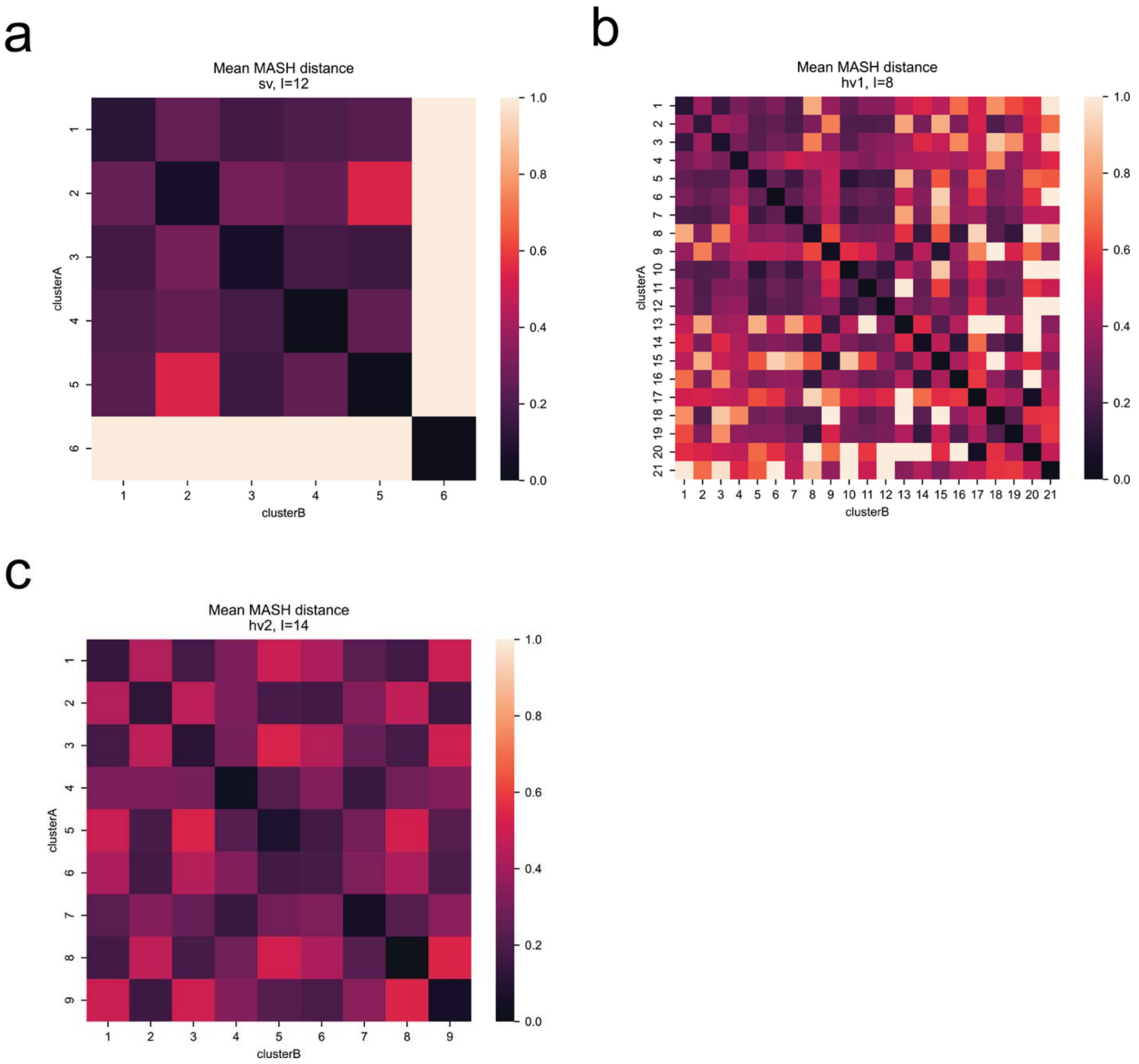

Figure S 11: Sequences generally are more similar within clusters than between clusters and clusters have distinct sequence motifs. The mean nucleotide distance between pairs of sequences in the same and different clusters in the semivariable (a), hypervariable 1 (b), and hypervariable 2 (c) regions.

bioRxiv preprint doi: https://doi.org/10.64898/2026.02.02.703239; this version posted February 5, 2026. The copyright holder for this preprint (which was not certified by peer review) is the author/funder, who has granted bioRxiv a license to display the preprint in perpetuity. It is made available under aCC-BY 4.0 International license.

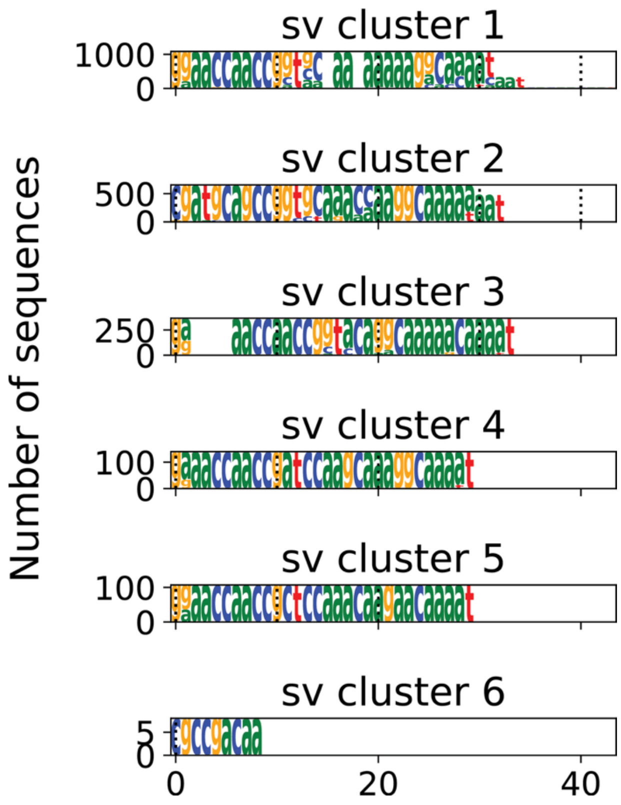

Figure S 12: The sequence logos for each cluster of the semivariable sequences. The nucleotide sequences were aligned using MAFFT in each cluster. The height of the nucleotides represents the number of sequences with each nucleotide.

bioRxiv preprint doi: https://doi.org/10.64898/2026.02.02.703239; this version posted February 5, 2026. The copyright holder for this preprint (which was not certified by peer review) is the author/funder, who has granted bioRxiv a license to display the preprint in perpetuity. It is made available under aCC-BY 4.0 International license.

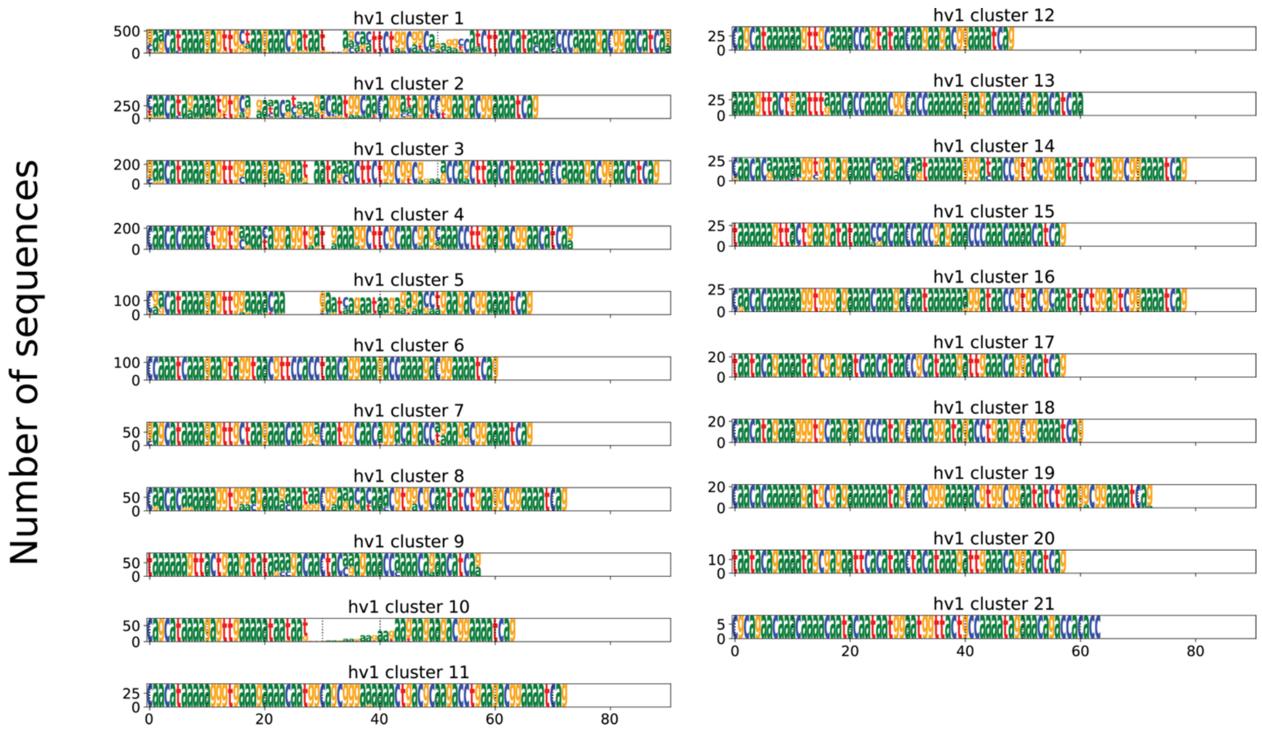

Figure S 13: The sequence logos for each cluster of the hypervariable 1 sequences. The nucleotide sequences were aligned using MAFFT in each cluster. The height of the nucleotides represents the number of sequences with each nucleotide.

bioRxiv preprint doi: https://doi.org/10.64898/2026.02.02.703239; this version posted February 5, 2026. The copyright holder for this preprint (which was not certified by peer review) is the author/funder, who has granted bioRxiv a license to display the preprint in perpetuity. It is made available under aCC-BY 4.0 International license.

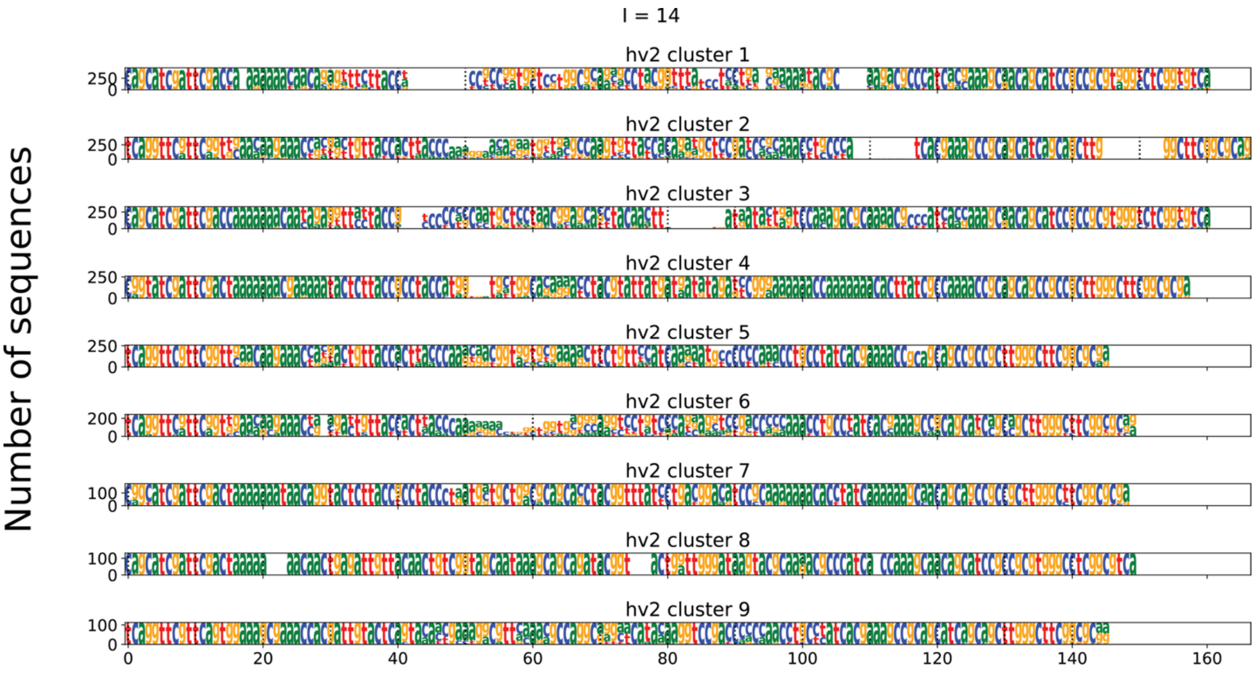

Figure S 14: The sequence logos for each cluster of the hypervariable 2 sequences. The nucleotide sequences were aligned using MAFFT in each cluster. The height of the nucleotides represents the number of sequences with each nucleotide.

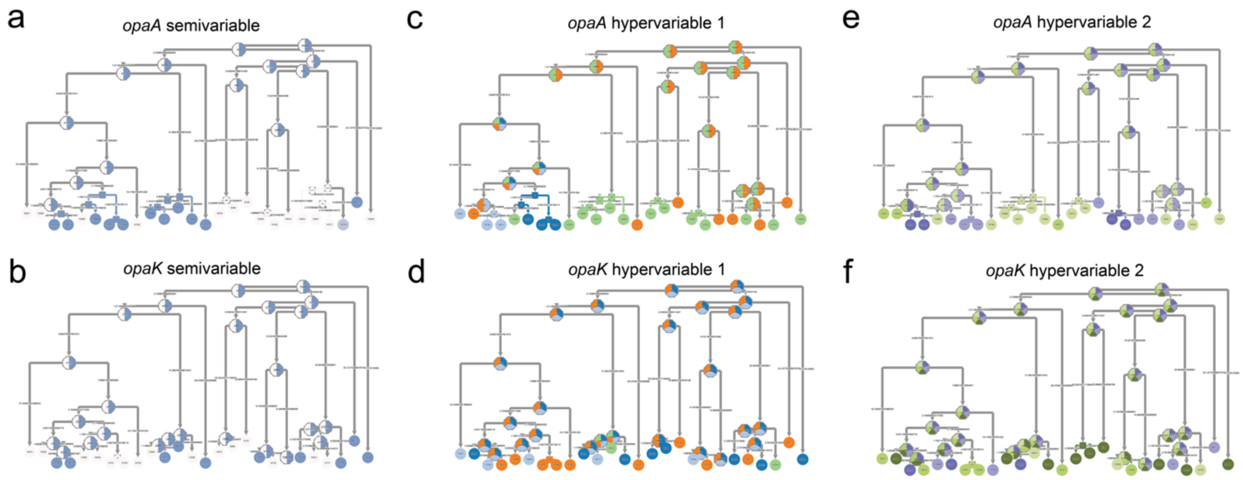

Figure S 15: *opaA* and *opaK* have similar cluster types that change at different rates. The dated phylogeny of the subtree shown in **Figure 6b** annotated with the cluster types for the

bioRxiv preprint doi: https://doi.org/10.64898/2026.02.02.703239; this version posted February 5, 2026. The copyright holder for this preprint (which was not certified by peer review) is the author/funder, who has granted bioRxiv a license to display the preprint in perpetuity. It is made available under aCC-BY 4.0 International license.

semivariable (a-b), hypervariable 1 (c-d), and hypervariable 2 (e-f) regions shown as colored circles for *opaA* (a, c, e) and *opaK* (b, d, f). The colors are comparable within each sequence region across loci (e.g., the same color scheme is used for the semivariable region of both *opaA* and *opaK*) but not between sequence regions. The colored circle annotations at the tips represent the cluster types of the isolates, and the colored circle annotations in the internal nodes represent the inferred ancestral state by PastML. The cluster types that are present in *opaA* and *opaK* are similar, but more closely related isolates have more similar cluster types in *opaA* compared to *opaK*.

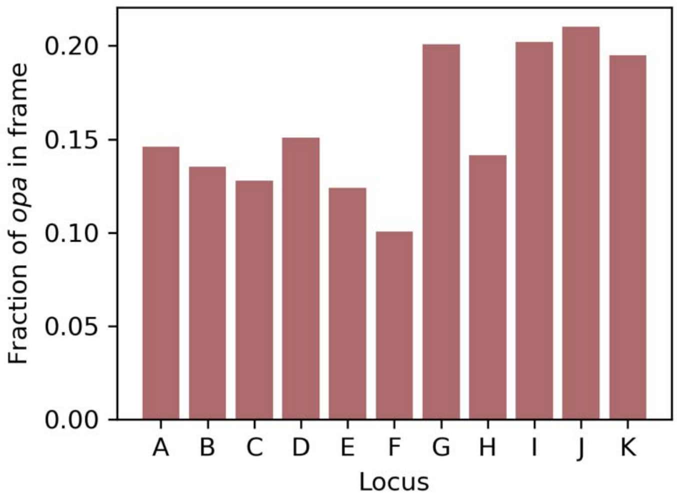

Figure S 16: No significant differences in fraction in of *opa* in frame by locus. One-sided proportions *Z*-tests with a Bonferroni multiple hypothesis correction comparing the fraction of *opa* in frame at each locus (1) to the total fraction of *opa* in frame across all loci and (2) to the fraction of *opaK* alleles that are in frame are not significant.

bioRxiv preprint doi: https://doi.org/10.64898/2026.02.02.703239; this version posted February 5, 2026. The copyright holder for this preprint (which was not certified by peer review) is the author/funder, who has granted bioRxiv a license to display the preprint in perpetuity. It is made available under aCC-BY 4.0 International license.

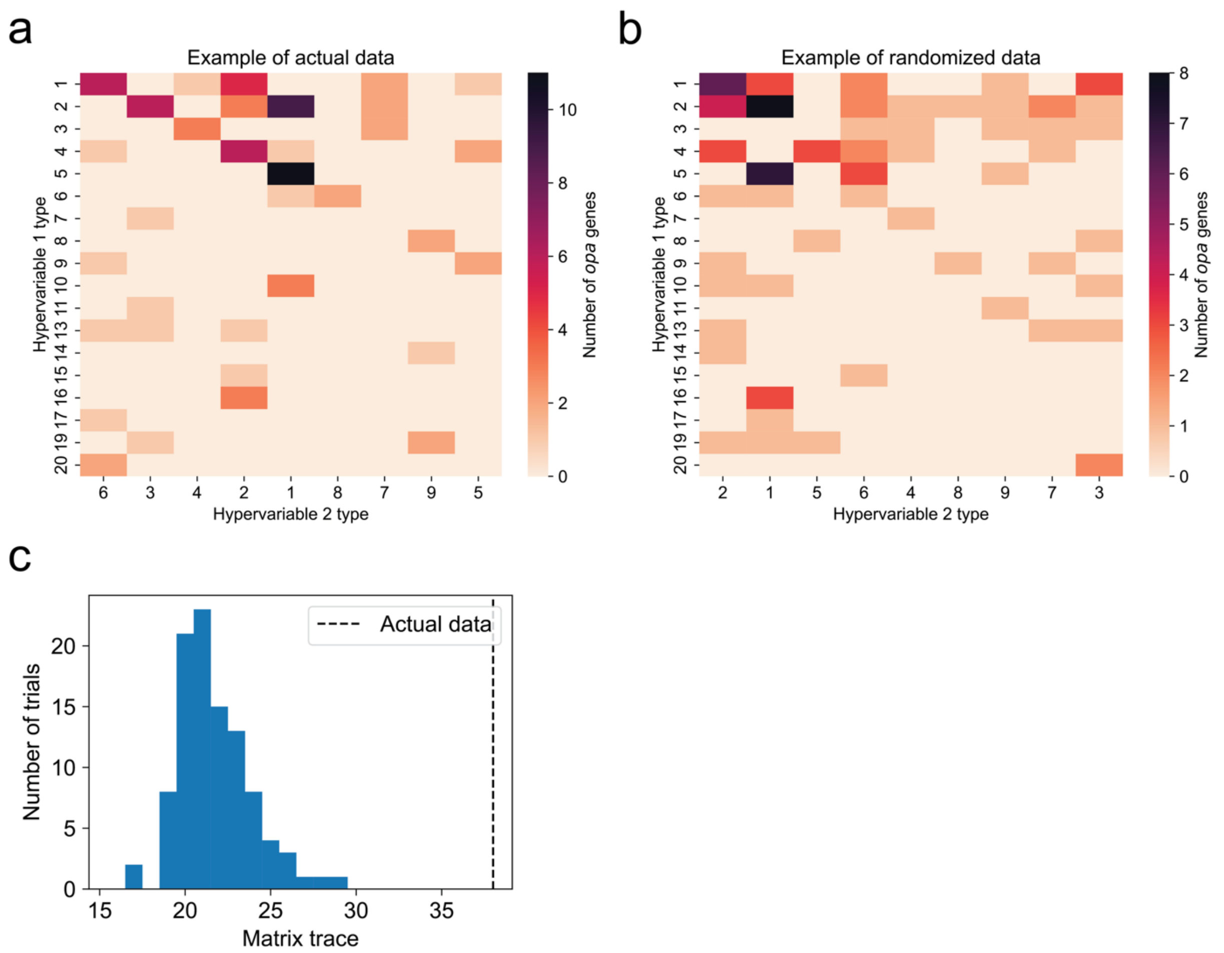

Figure S 17: Non-random association of hypervariable 1 and hypervariable 2 allele types in *opa* genes, as shown for one representative random subset of 1 isolate per BAPS cluster. (a) The number of each combination of hypervariable 1 and hypervariable 2 types in *opa* alleles after accounting for isolate sampling and population structure. The columns of the matrix were rearranged to give the largest matrix trace (padding with columns of all zeros to make a square matrix). (b) The same data representation in (a) but after randomizing the assignment of hypervariable 2 types across the *opa* alleles. (c) The distribution of the maximum sum of the diagonal matrix elements (allowing for column rearrangements) in 100 randomizations of the hypervariable 2 type (blue histogram) compared to the actual data (black dashed line).

bioRxiv preprint doi: https://doi.org/10.64898/2026.02.02.703239; this version posted February 5, 2026. The copyright holder for this preprint (which was not certified by peer review) is the author/funder, who has granted bioRxiv a license to display the preprint in perpetuity. It is made available under aCC-BY 4.0 International license.

Supplementary Information

Supplementary Methods

Short-read sequencing

Short-read sequencing for EEE029 was performed in Bristow, Mortimer, et al. [68] and short-read sequencing of UMASS-DGI_65 was performed using the same method. Briefly, DNA was extracted from bacterial cells grown overnight on GCB-K plates at 37°C with 5% carbon dioxide using the Invitrogen PureLink Genomic DNA Mini Kit. Libraries were prepped and sequenced on the Illumina NextSeq 2000 sequencer at the Microbial Whole Genome Sequencing Center or the Bauer Core Facility at Harvard University.

Comparison of long-read assembly and polishing methods

To develop a pipeline for assembling complete genomes to minimize errors in the *opa* genes, we used the following assembly and polishing approaches using the filtered long-reads from Oxford Nanopore (see Methods) and short reads generated by Illumina NextSeq: Flye v2.9.5 [27], Medaka v2.0.1 (https://github.com/nanoporetech/medaka), Polypolish v0.6.0 [69], Pypolca v0.3.1 [69], and Autocycler v0.2.1 [25]. We tested the following workflows:

1. Long-read only assembly with Flye.
2. Long-read only assembly with Flye → long-read polishing with Medaka
3. Long-read only assembly with Flye → long-read polishing with Medaka → short-read polishing with Polypolish default
4. Long-read only assembly with Flye → long-read polishing with Medaka → short-read polishing with Polypolish default followed by short-read polishing with Pypolca careful
5. Long-read only assembly with Autocycler

bioRxiv preprint doi: https://doi.org/10.64898/2026.02.02.703239; this version posted February 5, 2026. The copyright holder for this preprint (which was not certified by peer review) is the author/funder, who has granted bioRxiv a license to display the preprint in perpetuity. It is made available under aCC-BY 4.0 International license.

6. Long-read only assembly with Autocycler → short-read polishing with Polypolish default
7. Long-read only assembly with Autocycler → short-read polishing with Polypolish default → short-read polishing with Pypolca careful

We used the genomes UMASS-DGI_65 and EEE029 which had sequencing depth of around 140x. To test how sequencing depth would affect the *opa* sequence accuracy, we randomly subsampled the reads using the seqtk package v1.4 (https://github.com/lh3/seqtk) to 15% (21x), 30% (42x), and 60% (84x), and compared to the results including 100% of the reads (140x). For each read subset, we assembled the genome using the approaches above and identified the *opa* sequences as described in the Methods. We aligned the resulting *opa* sequences using MAFFT v7.520 [35] with the default parameters.

More extensive testing of Autocycler with different read depth across diverse isolates

We selected 11 phylogenetically diverse isolates with at least 125x coverage using Treemmer v0.3 [70]. We created 10 random read subsets using the seqtk package at 50x, 75x, 100x, and 125x coverage. We assembled the genomes using the following approaches. We assembled the genomes (using long reads only) using Autocycler and followed the approach above to compare *opa* sequences.

Algorithm to search for opa *genes in the complete genomes*

We wrote a custom python script to identify *opa* genes. First, we searched for 3 to 100 tandem repeats of the CTCTT pentanucleotide with at most 2 substitutions across the entire sequence, which we will refer to as the coding repeats (CR), similar to the approach used in Bilek et al. [21]. We then searched for the unique conserved sequence near the stop codon (TGCGCTACCGCTTCTGAT) with at most 2 substitutions, which we will refer to as the term sequence. Pairs of CR and term sequences were matched if they were separated by less than 1200 bp in the genome. For term sequences that were unpaired, a more lenient search was

bioRxiv preprint doi: https://doi.org/10.64898/2026.02.02.703239; this version posted February 5, 2026. The copyright holder for this preprint (which was not certified by peer review) is the author/funder, who has granted bioRxiv a license to display the preprint in perpetuity. It is made available under aCC-BY 4.0 International license.

performed for the upstream CR where we allowed up to 1 error for each CR unit (substitution, insertion, or deletion) to catch CR units that contained mutations. If there was still not a matching CR found, it could have been that the *opa* did not have a CR; in this case, we looked for the sequence found directly upstream of the CR consisting of a poly-A sequence of length 5-7 followed by CCTT and allowed one error (substitution, insertion, or deletion).

The start codon (ATG) was identified in the 50 bp upstream of the start of the CR sequence and the stop codon (TGA) was contained in the term sequence. Occasionally, the beginning and end of the CR region was not defined precisely with the above procedure due to errors in the CR (substitution, insertion, or deletion). Thus, the start of the CR was made more precise by finding the upstream sequence (see end of previous paragraph). If this sequence was not found, then the start of CR from earlier was used. Similarly, the end of the CR was made more precise by searching for the downstream sequence, which is CCG, allowing for the first C to be optional and 1 additional error.

Calculation of the randomness of hypervariable 1 and hypervariable 2 allele types

We dropped duplicate *opa* genes in the same genome as determined by having the same semivariable, hypervariable 1, and hypervariable 2 cluster type combinations to remove the effect of recent gene conversion events that have not yet had the time to be subject to selection.

We accounted for isolate sampling and *N. gonorrhoeae* population structure by partitioning the recombination-corrected phylogeny of complete genomes using fastbaps v1.0.8 [71] with the BAPS prior. We randomly selected 1 isolate from each BAPS cluster.

bioRxiv preprint doi: https://doi.org/10.64898/2026.02.02.703239; this version posted February 5, 2026. The copyright holder for this preprint (which was not certified by peer review) is the author/funder, who has granted bioRxiv a license to display the preprint in perpetuity. It is made available under aCC-BY 4.0 International license.

We determined the number of times each hypervariable 1 and hypervariable 2 cluster types appeared together in all *opa* genes in the subsampled isolates and created an association matrix where the rows were hypervariable 1 types, the columns were hypervariable 2 types, and the element values were the number of times each combination appeared. Because the number of hypervariable 1 and hypervariable 2 types were unequal, we padded columns with zeros to give a square matrix. We determined the degree of association by rearranging the columns of the association matrix to maximize the sum of diagonal entries using the Munkres algorithm (https://github.com/bmc/munkres?tab=readme-ov-file, v1.1.4). To determine whether the observed data could be random, we randomized the hypervariable 2 cluster types across the *opa* genes 100 times. We compared the maximum sum of diagonal elements from the observed data and the randomized data. We then repeated this process by performing 99 random subsamples of the isolates in each BAPS cluster.

Quantification of frameshift mutations downstream of the coding repeats leading to a premature stop codon

We identified all *opa* amino acid sequences that had a premature stop codon after the end of the coding repeat sequence. The approximate genome coverage was calculated as the number of input read bases divided by the number of consensus assembly bases. To identify the location of the frameshift mutations, the nucleotide sequences were aligned with MAFFT v7.520 [35] with the default parameters and visualized in Jalview v2.11.4.1 [36].

<u>Comparison of long-read assembly and polishing methods</u>

We first compared multiple assembly and polishing approaches, including Flye, Medaka, Polypolish, Pypolca, and Autocycler at 4 read depths (21x, 42x, 84x, and 140x) for two genomically diverse isolates. The *opa* sequences were identical across assemblies except for 1 SNP in UMASS-DGI_65 *opa7* and a 1 base insertion in EEE029 *opa11*, both of which occurred

bioRxiv preprint doi: https://doi.org/10.64898/2026.02.02.703239; this version posted February 5, 2026. The copyright holder for this preprint (which was not certified by peer review) is the author/funder, who has granted bioRxiv a license to display the preprint in perpetuity. It is made available under aCC-BY 4.0 International license.

in methods 1 (long-read assembly with Flye) and 2 (long-read assembly with Flye followed by long-read polishing with Medaka) for a read depth of 21x (**Figure S *1***). This analysis suggested Autocycler was the best performing assembly method and that polishing the Autocycler assemblies with short reads did not affect the *opa* sequences. However, because we only tested 2 genomes, we wanted to expand the analysis to include more isolates that are representative of *N. gonorrhoeae* diversity and to subset the reads to test lower read depths more systemically.

In our more extensive testing of Autocycler using 11 diverse isolates and 4 read depths (50x, 75x, 100x, and 125x) the *opa* sequences were identical across assemblies except for the following differences (always compared to 125x coverage assembly) (**Figure S *2***):

- EEE023 *opa4* had 1 SNP in 10/10 assemblies with 50x coverage.
- FFF007 *opa11* was not detected in 10/10 assemblies with 50x coverage due to a missing stop codon and had multiple sequence differences in 10/10 assemblies with 75x coverage.
- GCGS0313 *opa1* had a 1 base deletion in 9/10 assemblies with 125x coverage
- GCGS0313 *opa5* had 1 SNP in 10/10 assemblies with 50x coverage

Despite these changes in a small number of *opa* sequences, we concluded that most *opa* sequences were identical across read depths and that Autocycler assemblies were good enough for our purposes of looking at diversity and evolution across a large set of *opa* genes.

Supplementary Tables

Table S1. Publicly available complete genomes.

Table S2. Complete genomes sequenced in this study.

bioRxiv preprint doi: https://doi.org/10.64898/2026.02.02.703239; this version posted February 5, 2026. The copyright holder for this preprint (which was not certified by peer review) is the author/funder, who has granted bioRxiv a license to display the preprint in perpetuity. It is made available under aCC-BY 4.0 International license.

Table S3. References for publicly available *N. gonorrhoeae* short-read sequencing data meeting quality control thresholds for selection of representative draft genomes.

Table S4. Representative *N. gonorrhoeae* draft genomes.

Table S5. Publicly available *Neisseria* species complete genomes.
